# Healthcare Utilization Under a Comprehensive Public Welfare Program: Evidence From Japan

**DOI:** 10.3389/fpubh.2022.895679

**Published:** 2022-06-23

**Authors:** Michio Yuda

**Affiliations:** Graduate School of Economics and Management, Tohoku University, Sendai, Japan

**Keywords:** moral hazard, public assistance, outpatient, health care utilization, fixed-effect model, propensity score matching (PSM), Japan

## Abstract

The public assistance system in Japan provides detailed and comprehensive livelihood support for low-income families with various needs. As one example, and the beneficiaries of the public welfare program in Japan can receive the same medical treatments as those insured of the universal public health insurance without any financial burdens. This system has greatly contributed to maintaining and improving the health of public assistance beneficiaries but may cause excessive healthcare utilization: moral hazard. This study uses a large sample taken from two nationally representative claim data for public assistance and public health insurance patients to estimate the magnitude of moral hazard effect in basic outpatient utilization. The results of the fixed-effect regression analysis utilizing the concept of pseudo panel data analysis and those of propensity score matching show that the average treatment effect of public assistance assignment on healthcare utilization is significantly positive. Specifically, public assistance assignment increases monthly healthcare expenditure by 17.5 to 22.9 percent and the monthly number of doctor visits by 23.1 to 27.8 percent, respectively. In addition, the average treatment effects on the treated are also significantly positive, suggesting that monthly healthcare expenditure significantly decreases by 22.7 to 25.0 percent and the number of visits by 27.6 to 29.7 percent, respectively, when imposing a copayment on public assistance beneficiaries. However, the estimated price elasticity based on these results is very small, approximately −0.02, indicating that the level of copayment rate has little effect on the intensive margin of outpatient healthcare utilization.

## Introduction

In developed Organization for Economic Cooperation and Development (OECD) countries, the public welfare system mainly provides monetary benefits to low-income families, as a minimum livelihood security, and employment assistance for self-reliance. However, because low-income people are generally more susceptible to disease or lack adequate insurance, they postpone or avoid healthcare for economic reasons (1). In these countries, other public welfare systems provide them with healthcare services, and many empirical studies find that such social systems improve their access to healthcare (2–6) and contribute to improving their health and quality of life (7–11). On the contrary, some studies do not necessarily find such effects (12–14), and thus, the research results remain mixed.

**Figure 1:**
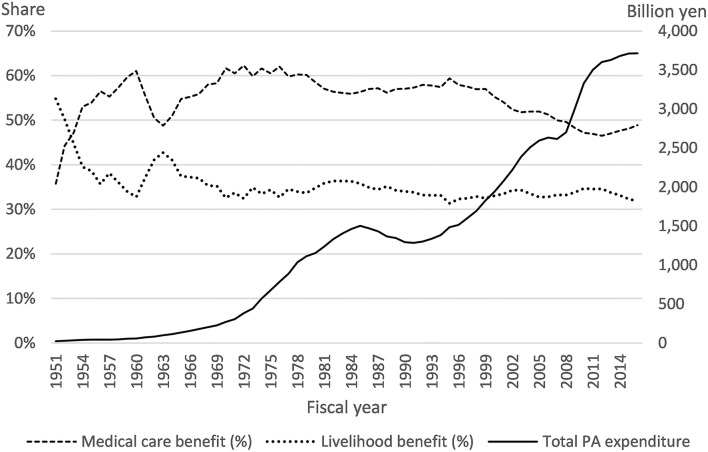
Trends in public assistance expenditures and shares of medical and livelihood benefits. Source from the *Annual Report on Social Security Statistics*, the General Administrative Agency of the Cabinet (1959–2000), and National Institute of Population and Social Security Research (2001–2016).

The public assistance (PA) system in Japan (*Seikatsu-Hogo Seido*) provides detailed and comprehensive livelihood support to low-income families with various needs, centering on income security, employment support, healthcare, long-term care, education, childbirth, funeral expenses, and housing. However, the system, which has existed for more than half a century, has several ongoing problems. First, as detailed in the next section, PA beneficiaries can receive the same medical treatments as those insured by the universal public health insurance (UPHI) without any financial burdens, such as insurance premiums, taxes, or copayments. Low-income people have worse health status and more healthcare needs than others, but it has also been pointed out that excessive healthcare utilization without any contribution to health leads to excessive healthcare expenditure, that is, moral hazard (15). In addition, because the share of healthcare costs to total PA expenditure has been the largest soon after the beginning of the system in 1950 (Figure 1), the Government Revitalization Unit has suggested that copayments for healthcare costs should be introduced to PA beneficiaries (16). On the contrary, the introduction of copayments may lead to a negative side effect of health deterioration by restraining the beneficiaries from visiting medical institutions. Therefore, it is important to examine how the PA system affects low-income people's healthcare utilization, but currently, there are only a few studies due to data unavailability. Second, because the PA system provides benefits within a lump-sum budget, it is difficult to operate a welfare system that provides comprehensive benefits in a severe and inflexible budget condition, such as that of Japan. In fact, the Japanese government has reduced the levels of livelihood benefits, the core benefit of the PA system, and additional benefits for certain family types, which has significantly influenced beneficiary families' consumption levels (17). Because only a few countries such as Finland, South Korea, Slovakia, and Sweden have a public welfare system providing comprehensive benefits (18), the empirical evidence for policy evaluation is decisively inadequate. Third, a PA system is one of the welfare policies, which also includes healthcare system for low-income population. In this regard, there is a potential concern that public officers in Japan who do not necessarily have medical knowledge may not be able to assess the PA beneficiaries' healthcare utilization correctly even if they attempt to improve the efficiency of healthcare system for the beneficiaries and to control their total healthcare expenditures.

In view of the above, this article uses large-scale individual data taken from the Japanese government's surveys to examine how outpatient healthcare utilization differs among PA beneficiaries and UPHI patients. Although several previous empirical studies have investigated that healthcare utilization in the Japanese PA system, to the best of our knowledge, no studies have focused on the basic outpatient healthcare services for PA beneficiaries. Therefore, the results of this study on how copayment level affects healthcare utilization among low-income people could provide useful policy implications for Japan's future PA system.

In addition, one of the major advantages of focusing on the Japanese PA system is that I can effectively control for some unobserved heterogeneity. Specifically, there is no local institutional heterogeneity in the Japanese PA system, unlike that observed, for example, in the U.S. Medicaid system. In addition, PA patients receive exactly the same medical treatment as public health insurance patients, and physicians, irrespective of their skill and experience, receive the same revenue by providing treatments to both PA and UPHI patients under the uniform national fee schedule. Moreover, there is no need to consider endogeneity between insurance choice and healthcare utilization, as in other countries, because the Japanese UPHI system is both universal and compulsory (19). These circumstances therefore remove common biases, which enable us to obtain consistent treatment effects.

The results from the fixed-effect (FE) estimation using the concept of pseudo panel data analysis and those of using propensity score matching (PSM) show that healthcare utilization by PA beneficiaries with no financial burden is significantly greater than that of those of UPHI patients, ceteris paribus. However, price elasticity is very low, approximately −0.02.

The rest of this paper is organized as follows: The next section presents our policy background and a discussion of our data and statistical models. Section Results provides descriptive statistics of the data, the main empirical results, and our robustness checks. I discuss our results and summarize our limitations in the last section.

## Materials and Methods

### Policy Backgrounds

The current PA system, based on the *Public Assistance Act* of 1950, is one of the oldest welfare programs in postwar Japan. This program provides comprehensive benefits such as employment support, healthcare, long-term care, education, childbirth, funeral expenses, and housing with income security at its core, to applicants who pass a means test administered by their prefectural or municipal government^1^ in order to maintain the minimum standards for wholesome and cultured living. All funding comes from taxes, with the central government contributing 75% and each local government 25%. There are some institutional differences in healthcare use between the PA and UPHI systems (Table 1). First, UPHI members contribute insurance premiums and taxes in advance, as well as copayments at a medical institution. In principle, PA patients can receive the same medical treatments without these financial burdens. On the contrary, the PA Act imposes access regulations on PA patients, requiring them to visit designated medical institutions with monthly healthcare and medicine vouchers that are issued by their local welfare office. This seems to contrast with the Japanese free access system for UPHI members, but in fact, there is little difference in terms of access, as PA beneficiaries receive their cash benefits and vouchers once a month at their welfare office, and approximately 90 percent of medical institutions accept both PA and UPHI patients. In addition, as medical fees for PA patients are reimbursed on the basis of the UPHI fee system, medical suppliers earn the same profit regardless of the type of patient. This means that the risk selection problem is unlikely to occur in Japan.

**Table 1:** Institutional differences in healthcare use between the public assistance and universal public health insurance systems in Japan.

	**Public assistance system**	**Public health insurance in 2007**
Financial source	Public funds (100%)	Premiums (49.0%), public funds (36.8%), and copayments and others (14.1%)
Copayment rate	0%	10% for those aged 70 and over^(a)^, 20% for those aged under 3, and 30% for all other enrollees^(b)^.
Patient access control	Visiting designated medical institutions by the *Public Assistance Act* and bringing monthly medical and medicine vouchers issued by the local welfare office.	None (free-access system)
Coverage	As for public health insurance	In-kind (90%) and cash benefit (10%)
Medical supply	Designated medical facilities under the *Public Assistance Act*	Designated medical facilities under the *Health Insurance Act* and the *National Health Insurance Act*.
Medical fee schedule	As for public health insurance	Nationally uniform medical fee schedule
Review of claims	Municipalities or local welfare offices	The Social Insurance Medical Fee Payment Foundation and the Federation of National Health Insurance Organizations.
Population share (in 2007)	1.21%	98.79% (universal health insurance)

(a) *30% for the persons aged 75 with more than a certain income.*

(b) *The high-cost medical treatment system subsidizes patient's copayments if the monthly copayment exceeds a certain level (See section Policy Backgrounds)*.

Some Japanese studies find that this PA system has greatly contributed to maintaining and improving the health of PA beneficiaries. For example, Kumagai (20) uses prefectural data and finds that the PA system has basically contributed to improving the health of PA beneficiaries at the macro-level. In addition, Hayashi (21) uses municipal data and a quantile regression model and finds that local allocation grant subsidies effectively respond to changing healthcare needs in rural areas. However, other studies note that the current PA system for more than half a century has certain flaws and some inefficiencies. Theoretically, exemption from copayment increases healthcare utilization by PA beneficiaries. This income effect is broadly confirmed in aggregated outpatient services (22), hospitalizations (23), public long-term care services (24), and dental services (25). One interpretation is that low-income people generally have a low socioeconomic status (SES), which is strongly correlated with low health conditions and increased healthcare use. However, the absence of any financial burdens may also cause excessive healthcare use, that is, moral hazard (15), resulting in a social loss of excessive healthcare expenses. For example, Fu and Noguchi (24) use individual claim data from long-term care and examine its moral hazard effect; they find that PA beneficiaries have significantly higher costs and days of care than persons with public long-term care insurance. This can be interpreted as the presence of moral hazard, but its effect is very small because the associated price elasticity is only approximately −0.1.

### Data

The main data sources in this study are two nationally representative claim records from 2003 to 2007, the *Fact-finding Survey on Medical Assistance* for PA patients and the *Survey of Medical Care Activities in Public Health Insurance* for UPHI patients. These surveys are conducted annually by the Ministry of Health, Labor and Welfare (MHLW) in Japan to obtain basic information for system administration. Both surveys are repeated cross-sectional surveys consisting of randomly selected medical claims (not patient level^2^). This analysis makes use of a number of common items contained in both surveys, specifically, the patient gender, age, prefecture of residence, monthly healthcare expenditure, actual number of visits of medical care, major diseases according to the International Statistical Classification of Diseases (ICD), and type of medical institution (hospital or clinic^3^).

Our sample contains only outpatient claim data for May to control for seasonal effects on utilization, while former claims are available from March to May (latter claims are available for May only). In addition, our sample does not include 50,762 claims that would be subject to the *high-cost medical care benefit* system (*Kogaku Ryoyo-hi Seido*). This system subsidizes patient copayments if the monthly copayment exceeds 267,000 yen (44,000 yen for those covered by the Elderly Health Care System from 1983 to 2008); the copayment rate for healthcare expenses above that amount falls to 1%. However, as such patients are exceptional cases, for example, those with a severe disease or requiring long-term treatment, the level of copayment has little impact on healthcare utilization.

Using claim data entails that I focus on the intensive margin of healthcare utilization. Various empirical health economics studies have shown that copayment level largely influences the extensive margin, choice of a doctor visit, but low-income people are more price elastic or have worse health conditions. In the former case, an increase in copayment rate would cause other negative side effects, such as health deterioration, by restraining PA beneficiaries from visiting medical institutions. There is thus a clear significance in investigating such impacts on the intensive margin of low-income populations' healthcare utilization.

### Statistical Model

The empirical equation in this study is specified as follows:


(1) $$\text{In }Y_{ijt}=\beta_{0}+\beta_{PA}PA_{ijt}+x_{ijt}\beta_{x}+z_{jt}\beta_{z}+\mu_{i}+\lambda_{j}+\tau_{t}+u_{ijt}$$

*Y*_*ijt*_ is monthly healthcare utilization, which contains healthcare expenditure adjusted for 2005 prices, and the number of a doctor visit for claim *i* issued in prefecture *j* at year *t*. PA is a dummy variable that equals one if a claim is for a PA beneficiary and zero otherwise. Significantly positive ${\hat{\beta }}_{PA}$ means that a PA patient uses more healthcare than a UPHI patient.

However, our interpretation of a significantly positive ${\hat{\beta }}_{PA}$ should be noted. First, this may reflect not only the effect of moral hazard due to the exemption from copayment but also the inherently poor health conditions of a low-income PA beneficiary. This means that simply estimating the value of ${\hat{\beta }}_{PA}$ as the moral hazard effect may overestimate its scale. To address this issue, I add a fixed effect μ_*i*_ to Equation [1] to distinguish these causal effects. In other words, because attributes, such as low SESs and the poor health of low-income people, can be interpreted as individual fixed effects in a panel data model, ${\hat{\beta }}_{PA}$ can be interpreted as the effect of excessive utilization due to moral hazard by adding a fixed-effect term to the empirical equation. However, because the data used in this study are repeated cross-sectional data, the model cannot strictly consider an individual-level fixed effect. Fortunately, because the surveys are nationally representative random sampling surveys, conducted every year, the average characteristics of the samples are similar from year to year (see Appendix 1). Based on these sample characteristics and a concept of pseudo panel data analysis (26), I use the mean of each specific group's healthcare utilization as a proxy for a fixed effect. Specifically, I use the one-year lagged mean healthcare expenses by attributes (PA beneficiary, gender, age, and prefectures), ${\bar{\mu }}_{it-1}$, as the proxy for the fixed-effect term.

Another problem with interpreting ${\hat{\beta }}_{PA}\;$is an endogenous problem due to institutional PA assignments. As PA assignment is not random but based on the result of a means test by a local government, Suzuki and Zhou (27) and Hayashi (28) find that PA ratios vary among local governments, depending on fiscal status and local socioeconomic circumstances. This means that the estimation results from an equation without local characteristics are positively biased because *PA*_*ijt*_ and error term *u*_*ijt*_ are correlated. To address this problem, Yuda (23) uses instrumental variable (IV) estimation, but these results may not be consistent because this identification relies on the non-linear specification in the first-stage regression. In this analysis, I add several local (prefectural) characteristics, **z**_*jt*_, as confounding factors to Equation [1]. Specifically, these include financial capability index (FCI), one-year lagged PA ratio, physician density, and unemployment rate. FCI is an index that indicates the fiscal condition of local governments. It is the average of the ratio of standard fiscal revenue to standard fiscal need demand over the past 3 years (29), and a higher value indicates greater reserved financial resources. Although local governments, which are the main implementers of the PA system, bear a financial burden of the system, their actual share is less than the statutory share (25 percent) because most of the local burden is subsidized by the local allocation grant (28). Nevertheless, Suzuki and Zhou (27) find that municipalities with financial difficulty are stricter when accepting applications to the PA system and their PA ratios are significantly lower. In the empirical model, I use prefectural FCI and the ratio of municipalities within a prefecture whose FCI is <0.5 because prefectures are common geographical information that is available in the surveys. In addition, the lagged PA ratio is a proxy for a fixed cost of PA administration in each prefecture, which is also related to the above. Physician density (the number of physicians per 100,000 people) is a proxy for local healthcare resources, represents the accessibility of healthcare services, and is considered to have a positive impact on healthcare utilization. However, the financial situation of local governments affects the development of the public healthcare supply system (human production factors, such as the number of doctors and nurses, and physical production factors, such as medical equipment) in a region (27), which ultimately affects the healthcare utilization rate and health status of local residents. Therefore, it may be a confounding factor in the present analysis. Finally, unemployment rate is a proxy for economic fluctuation. PA ratio generally increases during economic downturns, and Suzuki and Zhou (27) find that a local unemployment rate significantly increases the PA ratio. On the contrary, the business cycle has been found to significantly affect population health and healthcare utilization in several developed countries (30, 31). This means that economic fluctuation is a confounding factor on both PA ratio and healthcare utilization.

**x**_*ijt*_ contains the characteristics of patients and suppliers. Patient characteristics are dummy variables of gender (female), 98 age categories, and ICD codes for 119 main diseases. Supplier characteristic is a hospital dummy variable (see footnote 4), which captures differences in the number and quality of medical devices, as well as in the number of medical staff. λ_*j*_ is a prefectural fixed effect, which captures the differences in rigor of medical claim reviews (32) and other unobserved heterogeneities among prefectures, and τ_*t*_ is yearly fixed effect that captures the effect of biennial medical fee revisions and of comprehensive annual changes in macroeconomic and other socioeconomic circumstances. *u*_*ijt*_ is an error term, and I estimate clustering robust standard errors that allow serial correlation of subjects within prefectures (33).

As Equation [1] is compliant with a FE model, ${\hat{\beta }}_{PA}$ can be interpreted as the average treatment effect (ATE) of zero price on healthcare utilization. However, the estimated ATE may still include potential biases because the dataset does not include household attributes that determine PA eligibility, such as household income and assets, family structure, and working status. Fu and Noguchi (24), facing the same analytic problem, use PSM to balance the observable heterogeneity in PA eligibility. This study follows their strategy to confirm the robustness of our ATE estimation. The ATE estimated by PSM is *ATE* = *E*[*ATE*_*p*(_*X*__*i*_)_], where *ATE*_*p*(_*X*__*i*_)_ = *E*[*Y*_*i*_| *MA*_*i*_ = 1, *p*(*X*_*i*_)]−*E*[*MA*_*i*_ = 0, *p*(*X*_*i*_)] and *p*(*X*_*i*_) is the estimated propensity score (see Appendix 2). The PSM-ATE is the difference between outcomes conditioned on propensity score of treatment and control groups, and it reveals the hypothetical gain from treatment to a randomly selected member of the population when the treatment has universal applicability (34, 35). In addition, comparing this PSM-ATE with ${\hat{\beta }}_{PA}$ obtained from estimating Equation [1] can show how serious potential bias is. Another great advantage of applying PSM is to facilitate an estimation of the average treatment effect on the treated (ATT), defined as *ATT* = *E*[*ATE*_*p*(_*X*__*i*_)_| *MA*_*i*_ = 1]. The PSM-ATT can be obtained by matching an observation in the treatment group to that with a similar propensity score in the control group, and it reveals the average gain from treatment for the treated (34, 35). Specifically, the ATT in this paper can be interrupted as the difference between healthcare utilization at zero price and counterfactual utilization at a 10 percent copayment rate. In addition, by employing PSM, I impose a common support condition and apply one-to-one nearest neighborhood matching (1:1 NNM) within a caliper of 0.001 of propensity score. Once a UPHI claim is matched, it is therefore excluded from the sample (i.e., NNM without replacement), but I further apply the 1:1 NNM with replacement and one-to-five (1:5) NNM with replacement for our robustness checks.

## Results

### Descriptive Statistics

Table 2 presents the descriptive statistics and the results of our mean comparison tests. For PA claims, monthly healthcare expenditure and the number of doctor visits are 31.3 percent (3,357 yen) and 40.4 percent (0.7 visits) higher than those of UPHI, respectively. These trends are consistent with the RAND health insurance experiment (HIE) (7), where a higher copayment rate is associated with lower utilization. In addition, the mean age of PA claims is 1.2 years older, but the difference in the proportion of females is approximately zero. In terms of the distribution of major diseases, compared to UPHI patients, PA persons have more musculoskeletal and connective tissue diseases (4.7 percent points), more endocrine, nutritional, and metabolic diseases (4.0 pp), more cardiovascular diseases (3.1 pp), more digestive diseases (2.7 pp), fewer genitourinary diseases (6.3 pp), fewer mental and behavioral disorders (4.1 pp), and fewer diseases of the skin and subcutaneous tissue (3.4 pp). PA patients visit a hospital 16.1 percent more often, and their lagged mean healthcare expenses are 6.0 percent (1,015 yen) higher than those of UPHI.

**Table 2:** Descriptive statistics and mean comparison tests.

**Sample**	**All**		**PA patients**		**UPHI patients**		**Mean difference test**	
**Variables**	**Mean**	**Std.Dvi**	**Mean**	**Std.Dvi**	**Mean**	**Std.Dvi**	**Difference**	**Std.Err**
Dependent variables								
Monthly health care expenditure (thousand yen)	11.243	13.050	14.083	16.707	10.726	12.196	3.357**	(0.034)
Monthly number of doctor visits	1.925	2.176	2.545	3.112	1.812	1.937	0.732**	(0.006)
Individual attributes								
Public assistance (=1)	0.154	0.361	1.000	0.000	0.000	0.000		
Female (=1)	0.587	0.492	0.581	0.493	0.588	0.492	−0.007**	(0.001)
Age	57.757	23.665	58.846	20.041	57.559	24.261	1.286**	(0.044)
Lagged mean MHCE (fixed effect)	16.964	13.737	17.823	18.539	16.808	12.663	1.015**	(0.038)
Main disease^‡^								
Certain infectious and parasitic diseases (= 1)	0.043	0.203	0.037	0.188	0.044	0.206	−0.008**	(0.000)
Neoplasms (= 1)	0.039	0.194	0.032	0.177	0.040	0.197	−0.008**	(0.000)
Diseases of the blood and blood-forming organs and certain disorders involving the immune mechanism (= 1)	0.003	0.056	0.003	0.051	0.003	0.057	−0.001**	(0.000)
Endocrine, nutritional and metabolic diseases (= 1)	0.077	0.266	0.111	0.314	0.071	0.256	0.040**	(0.001)
Mental and behavioral disorders (= 1)	0.092	0.290	0.058	0.234	0.099	0.298	−0.041**	(0.001)
Diseases of the nervous system (= 1)	0.026	0.160	0.029	0.168	0.026	0.158	0.003**	(0.000)
Diseases of the eye and adnexa (= 1)	0.084	0.278	0.091	0.288	0.083	0.276	0.008**	(0.001)
Diseases of the ear and mastoid process (= 1)	0.015	0.122	0.016	0.126	0.015	0.122	0.001**	(0.000)
Diseases of the circulatory system (= 1)	0.185	0.388	0.211	0.408	0.180	0.384	0.031**	(0.001)
Diseases of the respiratory system (= 1)	0.102	0.303	0.101	0.301	0.103	0.303	−0.002**	(0.001)
Diseases of the digestive system (= 1)	0.052	0.222	0.074	0.263	0.048	0.213	0.027**	(0.001)
Diseases of the skin and subcutaneous tissue (= 1)	0.074	0.262	0.045	0.208	0.080	0.271	−0.034**	(0.000)
Diseases of the musculoskeletal system and connective tissue (= 1)	0.098	0.298	0.138	0.345	0.091	0.288	0.047**	(0.001)
Diseases of the genitourinary system (= 1)	0.081	0.273	0.028	0.165	0.091	0.288	−0.063**	(0.000)
Pregnancy, childbirth and the puerperium (= 1)	0.003	0.053	0.000	0.019	0.003	0.057	−0.003**	(0.000)
Certain conditions originating in the perinatal period (= 1)	0.000	0.017	0.000	0.012	0.000	0.017	0.000**	(0.000)
Congenital malformations, deformations and chromosomal abnormalities (= 1)	0.002	0.043	0.002	0.040	0.002	0.044	0.000**	(0.000)
Symptoms, signs and abnormal clinical and laboratory findings, not Elsewhere classified (=1)	0.013	0.113	0.012	0.108	0.013	0.114	−0.001**	(0.000)
Injury, poisoning and certain other consequences of external causes (= 1)	0.009	0.096	0.012	0.107	0.009	0.094	0.003**	(0.000)
Medical supply								
Hospital (=1)	0.321	0.467	0.457	0.498	0.296	0.457	0.161**	(0.001)
Prefectural macro conditions^§^								
Prefectural financial capability index^(a)^	0.573	0.263	0.610	0.276	0.566	0.260	0.044**	(0.001)
Low FCI municipality ratio^(a)^	0.437	0.309	0.416	0.324	0.441	0.306	−0.025**	(0.001)
Lagged prefectural PA ratio^(b)^	0.011	0.006	0.014	0.006	0.011	0.006	0.003**	(0.000)
Physician density (per 100,000 persons)^(c)^	207.870	38.321	217.626	37.621	206.094	38.179	11.531**	(0.080)
Unemployment rate (%)^(d)^	4.782	1.131	5.110	1.118	4.723	1.123	0.388**	(0.002)
Observations	1,698,857		261,546		1,437,311			

** *represents statistical significance at the 1 percent level.*

‡ *In the empirical analyses, 119 middle-classified main illnesses according to the ICD-10 are used, and the reference group is the disease described as “symptoms, signs, and abnormal clinical and laboratory findings, not classified elsewhere.”*

§ *Source of the prefectural aggregated variables are as follows:*

(a) *Annual Statistical Report on Local Government Finance, Ministry of Internal Affairs and Communications,*

(b) *Survey on Local Public Finance Conditions, Ministry of Internal Affairs and Communications,*

(c) *Survey of Physicians, Dentists and Pharmacists, Ministry of Health, Labor, and Welfare, and*

(d) *Labor Force Survey, Statistics Bureau, Ministry of Internal Affairs and Communications*.

### Main Results

Table 3 presents the estimation results of the effect of PA assignment on healthcare utilization and shows that PA assignment significantly increases *Y*, regardless of the model. This indicates that PA patients use more healthcare than those of UPHI, ceteris paribus. Models (i) and (ii) are the ATEs by a FE model of Equation [1], indicating that PA assignment significantly increases monthly healthcare expenditure by 17.5 percent ($=\exp\left({\hat{\beta }}_{PA}\right)-1$) and the number of doctor visits by 23.1 to 23.2 percent, respectively. Models (iii) to (viii) are the PSM estimation results, and ATEs and ATTs are quite similar in the matched samples. As explained in Appendix 3, all matched samples are more identical than the raw sample, and the mean and median biases of the 1:5 NNM are the smallest. Models (iii) to (v) are ATE results, indicating slightly larger effects on healthcare expenditure (19.1 to 22.9 percent) and a variation in the number of visits (22.0 to 27.8 percent). On the contrary, ATT estimates of Models (vi) to (viii) are larger than ATEs and suggest that monthly healthcare expenditure significantly decreases by 22.7 to 25.0 percent and the number of visits by 27.6 to 29.7 percent, respectively, when imposing a copayment on PA beneficiaries. Because the share of the amount of patients' copayment to total healthcare expenses during the study period is approximately 14 percent (36), price elasticity, based on our ATT estimates, ranges from −0.018 to −0.016, which is approximately one tenth of the gold standard estimate of the RAND HIE of −0.20 (7). These estimates suggest that a zero copayment rate for low-income people would have little moral hazard effect on the intensive margin of their healthcare utilization.

**Table 3:** Effect of PA assignment on healthcare utilization.

**Dependent variable**	**ln (HCE)**	**ln (Visits)**	***N***
ATE			
(i) FE (with **x**)	0.161**	0.208**	1,698,857
	(0.013)	(0.007)	
(ii) FE (with **x** and **z**)	0.161**	0.209**	1,698,857
	(0.013)	(0.007)	
(iii) PSM (1:1, noreplacement)	0.206**	0.245**	507,163
	(0.002)	(0.002)	
(iv) PSM (1:1, replacement)	0.174**	0.199**	1,698,263
	(0.005)	(0.003)	
(v) PSM (1:5, replacement)	0.183**	0.203**	1,698,263
	(0.004)	(0.002)	
ATT			
(vi) PSM (1:1, noreplacement)	0.206**	0.244**	507,163
	(0.002)	(0.002)	
(vii) PSM (1:1, replacement)	0.223**	0.260**	1,698,263
	(0.005)	(0.003)	
(viii) PSM (1:5, replacement)	0.204**	0.243**	1,698,263
	(0.003)	(0.002)	

*HCE and Visits stand for monthly healthcare expenditure adjusted for 2005 price and the numbers of a doctor visit, respectively.*

** *represents statistical significance at the 1 percent level. Clustering robust standard errors allowing for correlated residuals within prefectures are in parentheses*.

### Robustness Checks: Sub-sample Analyses

In this sub-section, I check the robustness of our results by attempting the same analysis using several subsamples and infer what the previous main results reflect.

#### Different Copayment Rates in the UPHI

In the Japanese UPHI, the copayment rate varies according to the age of the insured. The specific copayment rates during the study period are 10 percent for those aged 70 and over and for bedridden patients aged 65 and over, 20 percent for preschool children, and 30 percent for other insured parties. Figure S1 in Appendix 4 plots the means of *Y* by age and shows that these trends change after the ages of 18, 60, and 70. Regarding those aged 18 and under, although the statutory copayment rate ranges from 20 to 30 percent, their actual copayment rate is much lower or close to zero because their parents sustain them and because the prefectural and municipal governments subsidize their copayments (37). In addition, the statutory copayment rate for those aged from 19 to 69 is 30 percent, but for those aged 60 and over, the patterns of healthcare utilization may change; the major retirement age during the study period is 60^4^, and retirement increases opportunity costs (38–41). For those persons aged 70 and over, the copayment rate decreases to 10 percent (the Elderly Health Care System; EHCS), which changes their trends in healthcare utilization (42–44). In this subsection, I attempt the same analysis using four age groups to estimate how a difference in copayment rates affects healthcare utilization. In particular, the ATT estimates clearly show that healthcare utilization increases among PA patients when their copayment rate changes from zero to 10 or 30 percent.

Table 4 summarizes the estimation results of ATEs and ATTs and shows that PA assignment significantly increases *Y* in all models. For those aged 18 and under, there are few differences among any of the methods, and the ATT indicates that the monthly healthcare expenditure and number of visits for PA patients are 4.0 to 6.3 percent and 6.6 to 6.8 percent higher, respectively. Although these estimates are smaller than those of the main results (Table 3) and of the other groups, the differences in utilization are not considered a result of moral hazard but of the inherently poor health conditions of low-income PA patients because the actual copayment rates among both groups in this age range are almost zero. On the contrary, I can confirm that the larger the differences in the groups' copayment rates are, the larger the differences in their healthcare utilization. Specifically, Models (iv) and (viii) are the results for EHCS patients, and their ATT estimates indicate that an increase in the copayment rate from zero to 10 percent results in an 18.9 to 23.2 percent decrease in monthly healthcare expenditure and a 22.2 to 25.4 percent decrease in the number of visits. Moreover, the ATT estimates in Models (ii), (iii), (vi), and (vii) indicate that an increase in the copayment rate from zero to 30 percent results in a 24.3 to 27.8 percent decrease in monthly healthcare expenditure and a 30.3 to 33.8 percent decrease in the number of visits. However, because the price elasticities still remain low, ranging from −0.023 to −0.008, the moral hazard effect is very small among all age groups.

**Table 4:** Effect of PA assignment on healthcare utilization by age groups.

**Dependent variable**	**ln (HCE)**				**ln (Visits)**			
**Sample**	**(i) Under**	**(ii) Aged**	**(iii) Over**	**(iv) Over**	**(i) Under**	**(ii) Aged**	**(iii) Over**	**(iv) Over**
	**18**	**19 to 60**	**61 (non EHS)**	**61 (EHS)**	**18**	**19 to 60**	**61 (non EHS)**	**61 (EHS)**
Mean difference	0.044**	0.240**	0.191**	0.113**	0.028**	0.261**	0.243**	0.178**
	(0.005)	(0.003)	(0.004)	(0.004)	(0.004)	(0.003)	(0.003)	(0.003)
N	165,129	577,512	309,466	646,750	165,129	577,512	309,466	646,750
ATE								
(i) FE (with **x**)	0.057**	0.182**	0.184**	0.115**	0.047**	0.246**	0.244**	0.168**
	(0.015)	(0.017)	(0.016)	(0.010)	(0.006)	(0.007)	(0.011)	(0.008)
N	165,129	577,512	309,466	646,750	165,129	577,512	309,466	646,750
(ii) FE (with **x** and **z**)	0.057**	0.182**	0.184**	0.115**	0.047**	0.246**	0.244**	0.168**
	(0.015)	(0.017)	(0.016)	(0.010)	(0.006)	(0.007)	(0.011)	(0.008)
N	165,129	577,512	309,466	646,750	165,129	577,512	309,466	646,750
(iii) PSM (1:1, noreplacement)	0.056**	0.223**	0.220**	0.175**	0.065**	0.268**	0.266**	0.204**
	(0.006)	(0.004)	(0.004)	(0.004)	(0.004)	(0.003)	(0.003)	(0.003)
N	38,881	173,690	159,767	126,360	38,881	173,690	159,767	126,360
(iv) PSM (1:1, replacement)	0.059**	0.214**	0.199**	0.118**	0.064**	0.236**	0.244**	0.166**
	(0.017)	(0.009)	(0.008)	(0.008)	(0.014)	(0.006)	(0.005)	(0.006)
N	164,011	575,611	307,369	646,331	164,011	575,611	307,369	646,331
(v) PSM (1:5, replacement)	0.055**	0.215**	0.211**	0.130**	0.062**	0.233**	0.248**	0.170**
	(0.017)	(0.008)	(0.006)	(0.006)	(0.012)	(0.005)	(0.004)	(0.004)
N	164,011	575,611	307,369	646,331	164,011	575,611	307,369	646,331
ATT								
(vi) PSM (1:1, noreplacement)	0.056**	0.222**	0.221**	0.174**	0.064**	0.267**	0.266**	0.203**
	(0.006)	(0.004)	(0.004)	(0.004)	(0.004)	(0.003)	(0.003)	(0.003)
N	38,881	173,690	159,767	126,360	38,881	173,690	159,767	126,360
(vii) PSM (1:1, replacement)	0.040**	0.245**	0.242**	0.208**	0.065**	0.277**	0.291**	0.227**
	(0.012)	(0.007)	(0.010)	(0.008)	(0.008)	(0.004)	(0.006)	(0.005)
N	164,011	575,611	307,369	646,331	164,011	575,611	307,369	646,331
(viii) PSM (1:5, replacement)	0.061**	0.217**	0.222**	0.174**	0.066**	0.265**	0.270**	0.201**
	(0.008)	(0.005)	(0.006)	(0.006)	(0.005)	(0.003)	(0.004)	(0.004)
N	164,011	575,611	307,369	646,331	164,011	575,611	307,369	646,331

*HCE and Visits stand for monthly healthcare expenditure adjusted for 2005 price and the number of a doctor visit, respectively.*

** *represents statistical significance at the 1 percent level. Clustering robust standard errors allowing for correlated residuals within prefectures are in parentheses*.

#### Types of Medical Institutions

As mentioned in Section Policy Backgrounds, because the copayment rate for PA beneficiaries is zero and their free access to medical institutions is practically allowed, their preference of medical treatments should be examined to understand their excessive healthcare utilization. For example, some PA beneficiaries may choose a hospital with substantial amounts of large and expensive medical equipment so that they can receive more valuable treatments for free, but other beneficiaries may choose a clinic that is easy to access. On the contrary, in Japan, approximately 75 percent of physicians work for a hospital and earn a fixed salary irrespective of their workloads and outcomes, while the remaining 25 percent work in their own clinic, and their income basically depends on the number of patients. Therefore, doctors in clinics may have a greater financial incentive to overprovide medical services than those in hospitals (45). In this sub-section, I use these two subsamples to examine how the characteristics of medical suppliers affect PA patients' preferences for healthcare utilization.

Table 5 summarizes the estimation results of ATEs and ATTs and shows that PA assignment significantly increases *Y* in all models, but the effect on Y in clinics is larger than in hospitals.

**Table 5:** Effect of PA assignment on healthcare utilization by types of medical institutions.

**Dependent variable**	**ln (HCE)**		**ln (Visits)**	
**Sample**	**(i) Clinic**	**(ii) Hospital**	**(i) Clinic**	**(ii) Hospital**
Mean difference	0.284**	0.080**	0.296**	0.162**
	(0.002)	(0.003)	(0.002)	(0.002)
*N*	1,153,460	545,397	1,153,460	545,397
ATE				
(i) FE (with **x**)	0.220**	0.081**	0.242**	0.165**
	(0.011)	(0.019)	(0.008)	(0.007)
N	1,153,460	545,397	1,153,460	545,397
(ii) FE (with **x** and **z**)	0.220**	0.081**	0.242**	0.164**
	(0.011)	(0.018)	(0.008)	(0.007)
N	1,153,460	545,397	1,153,460	545,397
(iii) PSM (1:1, noreplacement)	0.286**	0.100**	0.281**	0.178**
	(0.003)	(0.004)	(0.002)	(0.002)
N	269,130	231,216	269,130	231,216
(iv) PSM (1:1, replacement)	0.243**	0.077**	0.240**	0.157**
	(0.006)	(0.007)	(0.004)	(0.004)
N	1,151,986	544,872	1,151,986	544,872
(v) PSM (1:5, replacement)	0.246**	0.066**	0.239**	0.155**
	(0.005)	(0.006)	(0.003)	(0.003)
N	1,151,986	544,872	1,151,986	544,872
ATT				
(vi) PSM (1:1, noreplacement)	0.286**	0.099**	0.281**	0.177**
	(0.003)	(0.004)	(0.002)	(0.002)
N	269,130	231,216	269,130	231,216
(vii) PSM (1:1, replacement)	0.303**	0.126**	0.296**	0.182**
	(0.006)	(0.008)	(0.004)	(0.004)
N	1,151,986	544,872	1,151,986	544,872
(viii) PSM (1:5, replacement)	0.291**	0.094**	0.283**	0.174**
	(0.005)	(0.005)	(0.003)	(0.003)
N	1,151,986	544,872	1,151,986	544,872

*HCE and Visits stand for monthly healthcare expenditure adjusted for 2005 price and the number of a doctor visit, respectively.*

** *represents statistical significance at the 1 percent level. Clustering robust standard errors allowing for correlated residuals within prefectures are in parentheses.*

*Hsealth Care Utilization under a Comprehensive Public Welfare Program: Evidence from Japan*.

Specifically, ATTs on monthly healthcare expenditure in hospitals range from 9.8 to 13.5 percent, but those in clinics range from 33.1 to 35.4 percent. In addition, the ATTs on the number of visits in hospitals range from 19.0 to 19.9 percent, but those in clinics range from 32.4 to 34.4 percent. These results suggest that PA outpatients prefer accessibility to a medical institution to receiving advanced medical services.

## Discussion

This study examines the impact of PA assignment on healthcare utilization using large individual datasets of PA and UPHI patients taken from two nationally representative claims data sets of the Japanese government. The results of the regression analysis utilizing a FE model based on the concept of pseudo panel data analysis and those of using PSM show that ceteris paribus, healthcare utilization by PA patients without a financial burden is higher than that of UPHI patients. However, the estimated price elasticity is very small at −0.02, indicating that the level of copayment rate has little effect on healthcare utilization in the intensive margin. Conversely, this result does not indicate an increasingly macro-trend of healthcare expenditure by PA beneficiaries, which implies that moral hazard effects would appear in the extensive margin.

One of the ongoing discussions on policy reform in the Japanese PA system is whether to introduce copayments to PA patients to reduce excessive healthcare expenditure due to the associated moral hazard (16). Because imposing a financial burden would discourage those PA beneficiaries who truly need medical care from visiting a doctor, its introduction is not without political difficulty and social criticism. However, our findings regarding the price inelastic intensive margin in healthcare utilization suggest that imposing a copayment for second and subsequent visits (reexaminations) could not only curb the overall moral hazard effect but also accommodate PA beneficiaries with worse health conditions. In addition, if policymakers prioritize controlling overall PA expenditure, it would be more effective to impose a regulation on their actual free access, as suggested by our results by the types of medical institution in Section Types of Medical Institutions that PA patients prefer accessibility to a medical institution. For example, a general and family practitioner in a clinic who has the basic clinical skills to deal with all diseases and health problems would be made as a gatekeeper for PA patients. Another would be to replace the current fee-for-service reimbursement system of the UPHI system with that of a DRG/PPS (Diagnosis Related Groups/Prospective Payment System) type that is specific to PA patients.

However, this work has some limitations. First, the increase in healthcare utilization due to the lack of a copayment from PA beneficiaries may be due not only to the inherently poor health conditions of low-income people and the moral hazard effect but also to supplier-induced demand (SID) (46). Specifically, PA patients are less likely to notice additional unnecessary and excessive medical treatment provisions because their actual financial burden is still zero. However, frequent SID provision is quite risky for medical suppliers in the long term because the MHLW can revoke the designation of a healthcare institution or the registration of an insured physician if fraudulent billing of healthcare expenditure is found to be intentional or grossly negligent. In addition, SID has little impact on healthcare expenditure because the costs of medical treatments are reimbursed to a medical institution only after they have been doubly reviewed by the public third-party payer and the insurer (32). In the extant empirical analyses, it is very difficult to identify each effect on inherently poor health, moral hazard, and SID, while the results of Yuda (23) provide useful evidence for an SID effect in the Japanese PA system. Yuda (23) focuses on short-term hospitalization that medical suppliers have broad discretion over healthcare provision and finds that its arc price elasticity is inelastic, only 0.2, which suggests that there is little effect of SID on healthcare expenditure in the Japanese PA system. Second limitation is that our claim data only include the intensive margin in healthcare utilization. There is room for discussion about what policy is most effective for controlling PA healthcare expenditure, depending on the estimates of price elasticity with respect to the extensive margin of healthcare utilization. Third, I do not evaluate how the PA system directly influences PA patients' health and utility or public health in society because our data do not include patient outcomes. For example, RAND HIE reports that low-income people imposed a certain copayment had increased hypertension, poorer vision, worse dental hygiene, and more serious conditions than the other group. It is also important to control for potentially confounding factors of individual heterogeneity (e.g., education, household income and asset, life habits, family structure, disease history, and disease severity) as well as medical supplier characteristics (e.g., number of staff and beds, available medical equipment, and management agency). In particular, the current PSM procedures do not control for economic conditions and attributes of the household that are used in the means test to determine PA eligibility, due to the lack of data. Thus, the estimation results can only reflect price elasticity to certain degree because conditional independence assumption may not be sufficiently satisfied. Hence, further analysis using other comprehensive data, including the above information, may help confirm the findings obtained in this analysis.

## Data Availability Statement

The data analyzed in this study is subject to the following licenses/restrictions: the Ministry of Health, Labor and Welfare (MHLW) in Japan for authorization allowed me to use the original survey data for this research under the Statistics Act (No. 53) pursuant to Article 33. To use the original individual data in this study, researchers need to submit their detailed research proposal to the MHLW in advance. Only after the MHLW approves their proposal, the researchers can access the data. Requests to access these datasets should be directed to the Ministry of Health, Labor, and Welfare in Japan, https://www.mhlw.go.jp/stf/toukei/goriyou/chousahyo.html (only Japanese pages exist). In addition, the sources of other prefectural data are listed in the paper.

## Author Contributions

The author confirms being the sole contributor of this work and has approved it for publication.

## Funding

This study was supported by research grants from the Japan Society for Promotion of Science (Nos. 26780180 and 18K01665).

## Conflict of Interest

The author declares that the research was conducted in the absence of any commercial or financial relationships that could be construed as a potential conflict of interest.

## Publisher's Note

All claims expressed in this article are solely those of the authors and do not necessarily represent those of their affiliated organizations, or those of the publisher, the editors and the reviewers. Any product that may be evaluated in this article, or claim that may be made by its manufacturer, is not guaranteed or endorsed by the publisher.

## Footnotes

^1^In general, urban inhabitants submit their PA applications to a municipal welfare office, whereas those in towns and villages submit theirs to a prefectural welfare office.

^2^In other words, the subject of this analysis is not patient unit. For example, if a patient visits more than one medical institution in a month, the system will generate medical claims for the number of institutions, and thus the same individual may be counted as a different individual. Unfortunately, due to data limitations, it is impossible to compile such cases into a single individual unit.

^3^Under the Japanese Medical Care Act, a *hospital* is a medical institution with more than 20 beds.

^4^*The Survey on Employment Conditions of Elderly Persons*, conducted every four years by the MHLW, reports that 91.1 percent of companies adopted a mandatory retirement age of 60 in 2004 and 85.2 percent in 2008.
