# Supplementary material for: Healthcare Utilization Under a Comprehensive Public Welfare Program: Evidence From Japan
Source: Front Public Health. 2022 Jun 23;10:895679. doi: 10.3389/fpubh.2022.895679 (PMC9259971; doi:10.3389/fpubh.2022.895679)
Supplement: Supplementary file 1 [file Data_Sheet_1.docx]

Online Appendices on Health Care Utilization under a Comprehensive Public Welfare Program: Evidence from Japan

Michio Yuda

Graduate School of Economics and Management

# Trends in Individual Attributes

Table A1 summarizes the descriptive statistics for each year by PA assignment. The distribution of each variable has been relatively stable without sudden changes.

# Estimation of Propensity Score

Table A2 shows the results of the logit model, estimating the probability of PA assignment for estimating the propensity score:

$$p\left( \mathbf{X}_{i} \right)=\Pr\left( {PA}_{i}=1 | \mathbf{X}_{i} \right)=\Lambda(\mathbf{X}_{i})$$

where $\mathbf{X}_{i}=\left( 1, \mathbf{x}_{ijt}, \bar{\mu}_{it-1}, \lambda_{j}, \tau_{t} \right)$ that are the same independent variables in Equation [1]: gender, dummy variables for age, main diseases, and hospital, lagged mean health care expenditure, yearly and prefectural fixed effects, and a constant term.

# Balance of the Variables in the Matched Sample

The important aspect of matching estimation is that the covariates between the treatment and control groups are balanced. The simplest way to ensure this is to conduct a mean difference test for each variable and to confirm its insignificance; however, recently, this method has been criticized on the following two points (A1, A2): First, its statistical significance depends on the sample size. When the sample size becomes large, as ours is, the statistical power becomes high. Thus, even if the mean difference between groups is actually small, it will be significant. Second, Imai et al. (A1) suggest that the issue of balance is entirely in-sample and therefore involves no inference to populations.

In this paper, I do not confirm the balances of each variable but compare the overall model properties between the original and matched samples. Table A3 shows that the pseudo R-squared of the matched samples is less than that of the raw sample and the mean and median biases of the matched samples are smaller than those of the raw sample. These statistics indicate that the matched samples are more balanced between PA and UPHI patients and that the one-to-five NNMs are the most homogeneous among them. In addition, the indicators of Rubin’s R and B that represents how the sample is sufficiently balanced indicate that all estimated Bs values exceed the reference value of 25, while all Rs values are within the reference range of [0.5, 2]. Regarding the p-values of the likelihood-ratio (LR) test, the joint insignificance of all the regressors are all significant, but this may be due to our large samples.

# Averages of Y, by Age for Each Group

Supplementary Figure 1 plots the averages of Y, by age for each group. The difference between the two groups is not large until the age of 18, when the Y of PA patients becomes clearly larger than that of UPHI participants. The health care expenditures of both types of patient peak around age 60, remain flat or increased slightly, and then decline after age 70. The number of doctor visits for PA patients changes in the same way, while that for UPHI patients increases until approximately age 70.

# References

A1. Imai K, King G, Stuart EA. Misunderstandings between experimentalists and observationalists about causal inference. J R Stat Soc (Stat Soc) (2008) 171:481-502. doi: 10.1111/j.1467-985x.2007.00527.x

A2. Austin PC. An Introduction to Propensity Score Methods for Reducing the Effects of Confounding in Observational Studies. Multivar Behav Res (2011) 46:399-424. doi: 10.1080/00273171.2011.568786

## Table A1 Trends in variables

| Sample | Beneficiaries | |  |  |  | Others |  |  |  |  |
| --- | --- | --- | --- | --- | --- | --- | --- | --- | --- | --- |
| Year | 2003 | 2004 | 2005 | 2006 | 2007 | 2003 | 2004 | 2005 | 2006 | 2007 |
| Dependent variables |  |  |  |  |  |  |  |  |  |  |
| Monthly health care expenditure (thousand yen in 2005 price) | 14.394 | 13.973 | 14.308 | 14.052 | 13.766 | 10.693 | 10.557 | 10.859 | 10.745 | 10.771 |
|  | (16.426) | (16.277) | (16.948) | (17.183) | (16.596) | (11.276) | (11.795) | (12.333) | (12.334) | (13.165) |
| Monthly number of doctor visits | 2.817 | 2.575 | 2.534 | 2.486 | 2.375 | 1.924 | 1.819 | 1.796 | 1.782 | 1.742 |
|  | (3.503) | (3.114) | (3.095) | (3.032) | (2.855) | (2.109) | (1.947) | (1.915) | (1.867) | (1.833) |
| Individual attributes |  |  |  |  |  |  |  |  |  |  |
| Public assistance (=1) | 1.000 | 1.000 | 1.000 | 1.000 | 1.000 | 0.000 | 0.000 | 0.000 | 0.000 | 0.000 |
|  | (0.000) | (0.000) | (0.000) | (0.000) | (0.000) | (0.000) | (0.000) | (0.000) | (0.000) | (0.000) |
| Female (=1) | 0.587 | 0.586 | 0.576 | 0.581 | 0.575 | 0.592 | 0.591 | 0.584 | 0.585 | 0.586 |
|  | (0.492) | (0.492) | (0.494) | (0.493) | (0.494) | (0.491) | (0.492) | (0.493) | (0.493) | (0.492) |
| Age | 58.478 | 58.949 | 58.581 | 59.036 | 59.099 | 56.551 | 57.387 | 57.832 | 57.695 | 58.327 |
|  | (20.020) | (19.889) | (20.118) | (20.120) | (20.032) | (24.451) | (24.259) | (24.155) | (24.265) | (24.137) |
| Lagged mean MHCE (fixed effect, thousand yen in 2005 price) | 17.830 | 18.032 | 17.299 | 18.034 | 17.913 | 16.447 | 16.054 | 16.857 | 17.466 | 17.168 |
|  | (20.548) | (16.949) | (17.556) | (18.747) | (18.834) | (12.577) | (11.401) | (13.199) | (13.241) | (12.689) |
| Primary disease |  |  |  |  |  |  |  |  |  |  |
| Certain infectious and parasitic diseases (= 1) | 0.033 | 0.035 | 0.039 | 0.038 | 0.038 | 0.045 | 0.049 | 0.044 | 0.043 | 0.042 |
|  | (0.179) | (0.184) | (0.194) | (0.190) | (0.191) | (0.207) | (0.216) | (0.205) | (0.202) | (0.201) |
| Neoplasms (= 1) | 0.032 | 0.032 | 0.031 | 0.033 | 0.034 | 0.038 | 0.038 | 0.041 | 0.041 | 0.043 |
|  | (0.176) | (0.175) | (0.173) | (0.179) | (0.181) | (0.192) | (0.191) | (0.199) | (0.198) | (0.204) |
| Diseases of the blood and blood-forming organs and certain disorders involving the immune mechanism (= 1) | 0.003 | 0.002 | 0.003 | 0.002 | 0.002 | 0.004 | 0.003 | 0.003 | 0.003 | 0.003 |
|  | (0.054) | (0.050) | (0.051) | (0.050) | (0.050) | (0.063) | (0.058) | (0.056) | (0.054) | (0.056) |
| Endocrine, nutritional and metabolic diseases (= 1) | 0.108 | 0.110 | 0.110 | 0.112 | 0.114 | 0.071 | 0.069 | 0.067 | 0.072 | 0.074 |
|  | (0.310) | (0.313) | (0.312) | (0.315) | (0.318) | (0.257) | (0.254) | (0.250) | (0.258) | (0.262) |
| Mental and behavioral disorders (= 1) | 0.047 | 0.051 | 0.057 | 0.067 | 0.064 | 0.067 | 0.097 | 0.111 | 0.109 | 0.107 |
|  | (0.212) | (0.220) | (0.232) | (0.250) | (0.245) | (0.251) | (0.297) | (0.314) | (0.311) | (0.309) |
| Diseases of the nervous system (= 1) | 0.028 | 0.028 | 0.025 | 0.033 | 0.031 | 0.020 | 0.022 | 0.024 | 0.029 | 0.033 |
|  | (0.164) | (0.165) | (0.155) | (0.178) | (0.173) | (0.140) | (0.148) | (0.152) | (0.168) | (0.179) |
| Diseases of the eye and adnexa (= 1) | 0.095 | 0.091 | 0.090 | 0.092 | 0.089 | 0.094 | 0.090 | 0.077 | 0.076 | 0.078 |
|  | (0.294) | (0.287) | (0.287) | (0.288) | (0.285) | (0.291) | (0.286) | (0.267) | (0.265) | (0.269) |
| Diseases of the ear and mastoid process (= 1) | 0.016 | 0.016 | 0.016 | 0.017 | 0.016 | 0.016 | 0.015 | 0.017 | 0.015 | 0.012 |
|  | (0.125) | (0.124) | (0.126) | (0.129) | (0.124) | (0.126) | (0.120) | (0.128) | (0.123) | (0.109) |
| Diseases of the circulatory system (= 1) | 0.215 | 0.219 | 0.213 | 0.206 | 0.205 | 0.181 | 0.177 | 0.179 | 0.182 | 0.180 |
|  | (0.411) | (0.413) | (0.409) | (0.404) | (0.404) | (0.385) | (0.382) | (0.383) | (0.386) | (0.384) |
| Diseases of the respiratory system (= 1) | 0.103 | 0.097 | 0.103 | 0.100 | 0.099 | 0.121 | 0.095 | 0.101 | 0.102 | 0.093 |
|  | (0.304) | (0.296) | (0.304) | (0.300) | (0.299) | (0.326) | (0.294) | (0.302) | (0.302) | (0.291) |
| Diseases of the digestive system (= 1) | 0.082 | 0.080 | 0.075 | 0.068 | 0.069 | 0.049 | 0.046 | 0.048 | 0.048 | 0.048 |
|  | (0.275) | (0.272) | (0.263) | (0.252) | (0.254) | (0.215) | (0.209) | (0.214) | (0.215) | (0.213) |
| Diseases of the skin and subcutaneous tissue (= 1) | 0.045 | 0.043 | 0.046 | 0.044 | 0.046 | 0.077 | 0.090 | 0.082 | 0.075 | 0.075 |
|  | (0.208) | (0.203) | (0.210) | (0.206) | (0.210) | (0.266) | (0.286) | (0.274) | (0.263) | (0.263) |
| Diseases of the musculoskeletal system and connective tissue (= 1) | 0.140 | 0.145 | 0.138 | 0.133 | 0.136 | 0.091 | 0.092 | 0.092 | 0.089 | 0.091 |
|  | (0.347) | (0.352) | (0.345) | (0.340) | (0.343) | (0.288) | (0.289) | (0.289) | (0.285) | (0.288) |
| Diseases of the genitourinary system (= 1) | 0.028 | 0.026 | 0.027 | 0.029 | 0.029 | 0.097 | 0.090 | 0.090 | 0.087 | 0.090 |
|  | (0.164) | (0.160) | (0.161) | (0.168) | (0.169) | (0.296) | (0.287) | (0.286) | (0.282) | (0.287) |
| Pregnancy, childbirth and the puerperium (= 1) | 0.000 | 0.000 | 0.000 | 0.000 | 0.000 | 0.005 | 0.004 | 0.002 | 0.002 | 0.002 |
|  | (0.021) | (0.020) | (0.019) | (0.018) | (0.018) | (0.072) | (0.062) | (0.046) | (0.050) | (0.050) |
| Certain conditions originating in the perinatal period (= 1) | 0.000 | 0.000 | 0.000 | 0.000 | 0.000 | 0.000 | 0.000 | 0.000 | 0.000 | 0.000 |
|  | (0.011) | (0.009) | (0.014) | (0.012) | (0.015) | (0.013) | (0.014) | (0.017) | (0.021) | (0.021) |
| Congenital malformations, deformations and chromosomal abnormalities (= 1) | 0.002 | 0.002 | 0.001 | 0.002 | 0.001 | 0.002 | 0.001 | 0.002 | 0.002 | 0.003 |
|  | (0.044) | (0.045) | (0.034) | (0.042) | (0.037) | (0.039) | (0.037) | (0.041) | (0.048) | (0.052) |
| Symptoms, signs and abnormal clinical and laboratory findings, not Elsewhere classified (=1) | 0.010 | 0.011 | 0.014 | 0.013 | 0.012 | 0.014 | 0.012 | 0.011 | 0.015 | 0.014 |
|  | (0.100) | (0.102) | (0.117) | (0.112) | (0.107) | (0.116) | (0.107) | (0.105) | (0.122) | (0.118) |
| Injury, poisoning and certain other consequences of external causes (= 1) | 0.011 | 0.012 | 0.012 | 0.011 | 0.012 | 0.008 | 0.009 | 0.010 | 0.008 | 0.010 |
|  | (0.105) | (0.108) | (0.109) | (0.105) | (0.109) | (0.089) | (0.093) | (0.098) | (0.090) | (0.099) |
| Medical supply |  |  |  |  |  |  |  |  |  |  |
| Hospital (=1) | 0.475 | 0.469 | 0.455 | 0.450 | 0.444 | 0.299 | 0.292 | 0.294 | 0.298 | 0.298 |
|  | (0.499) | (0.499) | (0.498) | (0.497) | (0.497) | (0.458) | (0.455) | (0.456) | (0.457) | (0.458) |
| Prefectural macro conditions |  |  |  |  |  |  |  |  |  |  |
| Prefectural financial capability index | 0.561 | 0.564 | 0.588 | 0.636 | 0.682 | 0.521 | 0.526 | 0.544 | 0.597 | 0.643 |
|  | (0.249) | (0.251) | (0.258) | (0.282) | (0.307) | (0.235) | (0.239) | (0.242) | (0.267) | (0.291) |
| Low FCI municipality ratio | 0.503 | 0.459 | 0.394 | 0.379 | 0.370 | 0.526 | 0.479 | 0.424 | 0.399 | 0.380 |
|  | (0.314) | (0.315) | (0.325) | (0.325) | (0.319) | (0.297) | (0.298) | (0.306) | (0.307) | (0.297) |
| Lagged prefectural PA ratio | 0.012 | 0.013 | 0.015 | 0.015 | 0.016 | 0.010 | 0.011 | 0.011 | 0.011 | 0.012 |
|  | (0.005) | (0.006) | (0.006) | (0.006) | (0.007) | (0.005) | (0.006) | (0.006) | (0.006) | (0.006) |
| Physician density (per 100,000 persons) | 211.982 | 214.623 | 217.375 | 219.790 | 222.648 | 202.419 | 203.677 | 205.256 | 207.908 | 211.197 |
|  | (37.379) | (37.945) | (36.868) | (37.348) | (37.639) | (37.498) | (37.925) | (37.859) | (38.273) | (38.701) |
| Unemployment rate (%) | 5.724 | 5.563 | 5.147 | 4.798 | 4.521 | 5.342 | 5.188 | 4.679 | 4.358 | 4.064 |
|  | (1.128) | (1.130) | (0.955) | (0.938) | (0.978) | (1.126) | (1.087) | (0.966) | (0.931) | (0.959) |
| Observations | 45226 | 49191 | 52799 | 55312 | 59018 | 281888 | 282019 | 291967 | 305399 | 276038 |

*Notes*: The values in the upper row are means and those in parentheses in the lower row are standard deviations.

## Table A2 Result of the logit model for estimating the propensity score

| Dependent variable | *Pr* (*PA*=1) |
| --- | --- |
| Female | 0.021** |
|  | (0.005) |
| Hospital | 0.556** |
|  | (0.005) |
| ln (lagged mean MHCE) | −0.362** |
|  | (0.002) |
| Constant | −2.717** |
|  | (0.123) |
| Age effects | Yes |
| Diseases effects | Yes |
| Year effects | Yes |
| Prefectural effects | Yes |
| Adjusted R-squared | 0.157 |
| Observations | 1,698,857 |

*Notes*: ** represents statistical significance at the 1 percent level. Clustering robust standard errors allowing for correlated residuals within prefectures are in parentheses.

## Table A3 Statistical indicators of the empirical samples

| Sample | Pseudo R-Squared | LR test | Mean bias | Median bias | Rubin's B statistics | Rubin's R statistics | %Var |
| --- | --- | --- | --- | --- | --- | --- | --- |
| Raw sample | 0.091 | 133466.27** | 5.0 | 4.3 | 78.4 | 1.1 | 100 |
| PSM (1:1, noreplacement) | 0.042 | 21196.57** | 2.8 | 1.6 | 50.1 | 1.4 | 100 |
| PSM (1:1, replacement) | 0.047 | 34121.04** | 3.3 | 2.1 | 51.9 | 1.2 | 100 |
| PSM (1:5, replacement) | 0.016 | 11847.62** | 1.8 | 1.3 | 30.3 | 1.3 | 100 |

*Notes*: ** represents statistical significance at the 1 percent level.


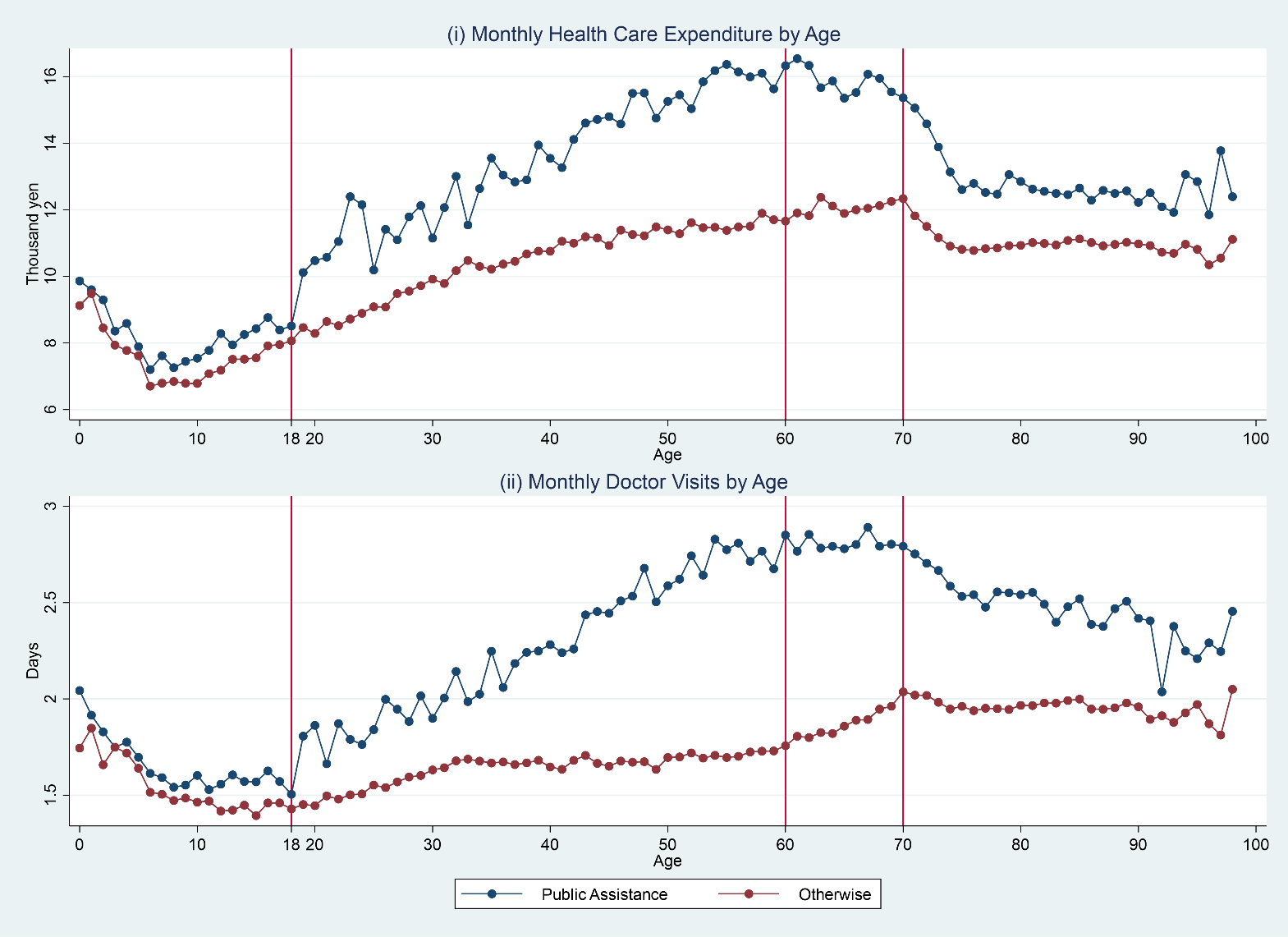


**Supplementary Figure 1.** Averages of health care utilization, Y, by age for each group.
